# Evaluation of QUADAS, a tool for the quality assessment of diagnostic accuracy studies

**DOI:** 10.1186/1471-2288-6-9

**Published:** 2006-03-06

**Authors:** Penny F Whiting, Marie E Weswood, Anne WS Rutjes, Johannes B Reitsma, Patrick NM Bossuyt, Jos Kleijnen

**Affiliations:** 1 MRC Health Services Research Collaboration, Department of Social Medicine, Canynge Hall, Whiteladies Road, Bristol, UK; 2 Centre for Reviews and Dissemination, University of York, UK; 3 Department of Clinical Epidemiology and Biostatistics, Academic Medical Center, University of Amsterdam, The Netherlands

## Abstract

**Background:**

A quality assessment tool for diagnostic accuracy studies, named QUADAS, has recently been developed. Although QUADAS has been used in several systematic reviews, it has not been formally validated. The objective was to evaluate the validity and usefulness of QUADAS.

**Methods:**

Three reviewers independently rated the quality of 30 studies using QUADAS. We assessed the proportion of agreements between each reviewer and the final consensus rating. This was done for all QUADAS items combined and for each individual item. Twenty reviewers who had used QUADAS in their reviews completed a short structured questionnaire on their experience of QUADAS.

**Results:**

Over all items, the agreements between each reviewer and the final consensus rating were 91%, 90% and 85%. The results for individual QUADAS items varied between 50% and 100% with a median value of 90%. Items related to uninterpretable test results and withdrawals led to the most disagreements. The feedback on the content of the tool was generally positive with only small numbers of reviewers reporting problems with coverage, ease of use, clarity of instructions and validity.

**Conclusion:**

Major modifications to the content of QUADAS itself are not necessary. The evaluation highlighted particular difficulties in scoring the items on uninterpretable results and withdrawals. Revised guidelines for scoring these items are proposed. It is essential that reviewers tailor guidelines for scoring items to their review, and ensure that all reviewers are clear on how to score studies. Reviewers should consider whether all QUADAS items are relevant to their review, and whether additional quality items should be assessed as part of their review.

## Background

QUADAS is a tool to assess the quality of diagnostic accuracy studies included in systematic reviews. We defined quality as being concerned with both the internal and external validity of a study. QUADAS was developed in a systematic manner, based upon three reviews of existing evidence and a Delphi procedure involving a panel of experts in diagnostic research [1]. Like all quality assessment tools, QUADAS is a measurement, implying that its characteristics have to be evaluated: does it measure what it aims to measure, how well does it do this, and are results reproducible between different observers [2]? The objective of this study was to evaluate QUADAS by determining agreement between reviewers and the consensus rating and variability among raters, and gathering feedback on reviewers' experiences of using QUADAS.

## Methods

### Assessment of the consistency and reliability of QUADAS

Three reviewers were asked to use QUADAS to independently rate the quality of 30 studies as part of a systematic review on the diagnosis of peripheral arterial disease. One QUADAS item, the use of an appropriate reference standard, was not assessed as studies were only included in the review if they used a specified reference standard.

The three reviewers had different backgrounds and levels of experience. Reviewer 1 had previously carried out several diagnostic systematic reviews and had used QUADAS; she also had a background in primary diagnostics. Reviewer 2 was a new reviewer – this was the first review that she had worked on, but she had previously worked in primary diagnostics. Reviewer 3 was an experienced reviewer who had worked on a number of systematic reviews. This combination of reviewers with was chosen to reflect the spectrum of likely QUADAS users.

A limited amount of information specific to the diagnosis of peripheral arterial disease was provided to help with the scoring of QUADAS, this applied to items 1 (spectrum composition), 4 (disease progression bias), and 12 (availability of clinical information). For all other items, the guidelines on scoring provided in the QUADAS background document were briefly summarised [3]. Although reviewers did have access to the background document they were not specifically requested to read this or use it when assessing study quality.

Our main interest was in the amount of agreement between the rating of each reviewer and the consensus rating, calculated as the proportion of studies for which each reviewer agreed with the consensus rating. In addition, we also examined inter-observer variability by calculating the kappa statistic. Both analyses were carried out for all QUADAS items combined and for each individual item. We chose to focus on the proportion of agreements between reviewers and the final consensus, as kappas can be misleading in certain circumstances [4].

### Piloting QUADAS in ongoing reviews

Reviewers who had used QUADAS in their reviews completed a short structured questionnaire asking how they used QUADAS and what their opinions of its usefulness were. Details of the questionnaire are provided in Table 1. A narrative synthesis was used to summarise results.

**Table 1:** Questionnaire for evaluation of QUADAS

a) Review details
b) Content of the tool:
• Did QUADAS cover all important items?
• Were any QUADAS items omitted, added or modified?
c) Background document:
• Was the background document easy to understand?
• Were scoring instructions understandable?
• Should any items have been scored differently?
d) Technical points
• How long did it take to complete QUADAS?
• Was inter-rater reliability assessed?
e) Overall conclusions
• Reviewers were asked to rate coverage, ease of use, clarity of instructions, and validity (whether QUADAS helped to distinguish between studies of different qualities) on a five point scale
• Would you us QUADAS again?
f) Additional questions
• How were the results of quality incorporated into the review?
• Was a training session organised to ensure reviewers applied the tool consistently?
• Reviewer details, including age, experience, professional background
• Have you previously been involved in the quality assessment of studies included in a systematic review?
g) Final comments

## Results

### Assessment of the consistency and reliability of QUADAS

Table 2 summarises the agreement between reviewers. Agreement between reviewers 1 and 2 and the final consensus rating was very good at 91 and 90%, and was slightly lower (85%) for reviewer 3. Overall reviewer variability was good [5] with a kappa of 0.65.

**Table 2:** Overall agreement between reviewers and agreement with consensus for each of the QUADAS items and for all items combined

**QUADAS item**		**Agreement with consensus diagnosis (%) (95% confidence interval)**			Reviewer variability (κ) (95% confidence interval)
			
		1	2	3	
All items combined		91 (88–94)	90 (86–93)	85 (81–89)	0.66 (0.63 to 0.67)

1	Was the spectrum of patients representative of the patients who will receive the test in practice? (spectrum composition)*	90 (73–98)	87 (69–96)	83 (65–94)	0.73 (0.60 to 0.76)
2	Were selection criteria clearly described? (selection criteria)	90 (73–98)	83 (65–94)	73 (54–88)	0.55 (0.33 to 0.61)
*3*	*Is the reference standard likely to correctly classify* the target condition? (reference standard)*				
4	Is the time period between reference standard and index test short enough to be reasonably sure that the target condition did not change between the two tests? (disease progression bias)*	87 (69–96)	90 (73–98)	83 (65–94)	0.68 (0.63 to 0.86)
5	Did the whole sample or a random selection of the sample, receive verification using a reference standard of diagnosis? (partial verification)	87 (69–96)	90 (73–98)	93 (78–99)	0.27(-0.06 to 0.39)
6	Did patients receive the same reference standard regardless of the index test result? (differential verification)	97 (83–100)	97 (83–100)	97 (83–100)	0.31 (-0.01 to 0.46)
7	Was the reference standard independent of the index test (i.e. the index test did not form part of the reference standard)? (incorporation bias)	100 (88–100)	100 (88–100)	93 (78–99)	-0.02 (-0.03 to -0.01)
8	Was the execution of the index test described in sufficient detail to permit replication of the test? (index test execution)	97 (83–100)	100 (88–100)	87 (69–96)	0.60 (0.33 to 0.73)
9	Was the execution of the reference standard described in sufficient detail to permit its replication? (reference standard execution)	93 (78–99)	93 (78–99)	93 (78–99)	0.81 (0.60 to 0.87)
10	Were the index test results interpreted without knowledge of the results of the reference standard? (test review bias)	90 (73–98)	87 (69–96)	97 (83–100)	0.55 (-0.04 to 0.75)
11	Were the reference standard results interpreted without knowledge of the results of the index test? (reference standard review bias)	93 (78–99)	93 (78–99)	93 (78–99)	0.68 (0.46 to 0.76)
12	Were the same clinical data available when test results were interpreted as would be available when the test is used in practice? (clinical review bias)*	90 (73–98)	93 (78–99)	50 (31–69)	0.18 (-0.13 to 0.36)
13	Were uninterpretable/ intermediate test results reported? (uninterpretable test results)	83 (65–94)	70 (50–85)	87 (69–96)	0.32 (0.18 to 0.44)
14	Were withdrawals from the study explained? (withdrawals)	90 (73–98)	83 (65–94)	80 (61–92)	0.38 (0.33 to 0.51)

* Items for which review specific details were added to QUADAS

^+^The item relating to reference standard was not assessed for this review

Agreement between reviewers and the final consensus rating was over 80% for all but four items: selection criteria, availability of clinical information, uninterpretable test results and withdrawals. The poor agreement for the availability of clinical information was related to reviewer 3 who had a very poor level of agreement (50%) with the final consensus rating; the other reviewers showed over 90% agreement with the final consensus. This suggests that reviewer 3 was interpreting this item differently to the other reviewers. The other three items, selection criteria, uninterpretable results and withdrawals, showed moderate agreement between each reviewer and the consensus rating suggesting that there may be difficulties in applying these items.

#### Piloting QUADAS in ongoing reviews

Twenty reviewers used QUADAS in their reviews and provided feedback via the structured questionnaire (Table 3). Fifteen reviewers came from the UK, two from Australia, two from the Netherlands, and one from Switzerland. Of those from the UK, seven were employees of the Centre for Reviews and Dissemination (CRD), which is where some of the researchers who developed QUADAS were based. The topics covered by the reviews included the diagnosis of: tuberculosis, urinary tract infection in children, haematuria, Dengue fever, prostate cancer, shoulder pain, epilepsy seizure focus, angina and myocardial infarction, infected diabetic foot ulcers, bacterial infections, lumbar fusion, multiple sclerosis, and osteoporosis. Diagnostic tests under evaluation included laboratory tests, imaging and physical examination. The number of studies included in the reviews ranged from 1 to 208 (median 28).

**Table 3:** Summary of responses to the questionnaire on reviewers' experience of using QUADAS

**Reviewer Number**	**Coverage?**	**Omit items?**	**Add items?**	**Modify item?**	**Easy to understand?**	**Scoring?**	**Different scoring?**	**Time to complete (minutes)**	**Inter-rater reliability?**	Coverage*	Ease of use*	Clarity of instruction*	Validity*	Use again?*
1	+	-	+	-	+	+	-	30–60	-	5	3	5	5	+
2	+	-	-	+	+	+	-	<10	-	4	4	3	4	+
3	+	+	-	-	+	+	-	<10	-	4	4	5	3	+
4	+	-	-	-	+	+	-	30–60	-	4	4	4	4	+
5	+	-	-	-	+	+	-	15–30	-	5	4	5	5	+
6	+	-	-	-	+	+	-	15–30	-	5	3	4	4	+
7	+	-	-	-	+	-	-	15–30	+	4	4	2	2	+
8	+	-	-	-	+	-	-	15–30	+	4	4	3	3	+
9	-	-	+	-	+	+	+	>60	-	4	5	5	4	+
10	+	-	-	-	+	+	-	<10	-	5	5	5	5	+
11	+	-	+	-	+	+	-	15–30	-	4	3	4	3	+
12	-	-	-	-	+	+	-	15–30	-	5	3	5	3	+
13	+	+	+	-	-	-	-	10–15	-	4	3	3	5	+
14	+	+	-	-	+	+	-	10–15	-	4	4	4	4	+
15	+	-	-	-	+	+	-	10–15	-	5	4	4	4	+
16	+	-	-	-	+	+	-	10–15	-	4	3	4	4	+
17	+	-	-	-	+	+	-	<10	-	4	4	4	4	+
18	+	-	-	-	+	+	-	<10	-	5	5	5	3	+
19	+	-	-	-	+	+	-	10–15	-	4	4	4	4	+
20	+	-	-	-	+	+	-	15–30	-	5	4	5	4	+

*Each of these items were rated from 1–5 where 1 is strongly disagree (very poor) and 5 is strongly agree (excellent)

#### Content of tool

The feedback from 20 reviewers on the content of the tool was generally positive: eighteen reviewers thought that QUADAS covered all important items, seventeen did not omit any items, sixteen did not add any items, and nineteen did not modify any items.

Two reviewers thought that QUADAS did not cover all important items, one felt that it did not adequately cover population characteristics (description of spectrum, age, setting, prevalence), that questions regarding therapy, the positivity threshold of test results, and study design should have been included as separate items. These comments were mainly related to the desire to have information on these items so that they could be explored in subgroup analysis. The other reviewer thought that the tool should cover whether data could be extracted into a 2 × 2 table.

Three reviewers omitted items from QUADAS. One stated, "on occasions there were no withdrawals". One reviewer omitted items on: reference standard, disease progression bias, partial verification bias, differential verification bias and incorporation bias as these were not applicable to the topic area because there was no reference standard (the review was on prostate biopsies). The other reviewer omitted the item relating to disease progression bias as this did not apply to studies included in their review. Another reviewer stated that they did not omit any items but that as most of the studies included in their review were diagnostic case control studies, items on the availability of clinical information and withdrawals were difficult to answer, and in most cases the issue of follow-up was not relevant.

Four reviewers added items to QUADAS: one added clinically relevant items specific to their review, one added "Do you have plans to characterise data which are unsuitable for primary analysis?", one added "Was the raw data available?" and one added a number of items relating to the availability of 2 × 2 data, confidence intervals, a description of the index and reference tests and a description of the test threshold.

One reviewer modified the items on uninterpretable results and withdrawals to add a "not appropriate" response. She stated that if there were no uninterpretable test results it was unclear how to rate this item.

#### Background document

All but one reviewer found the background document easy to understand, two did not understand the scoring guidelines, and one reviewer thought that the items concerning differential and partial verification bias should have been scored differently. One reviewer found the item on disease progression bias difficult to understand. However, this difficulty appeared to be related to how to score this item specifically for their review rather than a problem with the instructions provided in the background document. Two reviewers stated that they added topic specific information to the background document to help determine exactly how to score items for their review.

Despite efforts to keep the wording of QUADAS simple to increase international applicability, two non-native English speakers had some difficulty in understanding the QUADAS background document. They found the item on the availability of clinical information difficult to understand and did not know what was meant by uninterpretable or indeterminate data or results, and felt that the background document did not clarify this. In future revisions, clarity of phrasing will be a key consideration.

The reviewer who thought items should have been scored differently felt that the items relating to verification bias should have been formulated differently and suggested "was verification bias avoided? (i.e. did the whole sample or a random selection of the sample receive verification using a reference standard)".

#### Technical points

The time taken to complete QUADAS ranged from less than 10 minutes to over an hour. Five reviewers reported that it took them <10 minutes, five that it took 10–15 minutes, seven that it took 15 to 30 minutes, two that it took 30 to 60 minutes and one that it took more than an hour. Some of the reviewers included the time to read the whole paper and carry out data extraction and completing QUADAS in this time, whereas others only included the time taken to complete QUADAS. None of the reviewers assessed inter-rater reliability.

#### Overall conclusions

Reviewers' ratings of QUADAS for coverage, ease of use, clarity of instructions and validity were generally good, especially for coverage, which was rated as good or very good by all reviewers, and ease of use, which was rated as at least average by all reviewers. One reviewer rated the clarity of instructions and the validity of QUADAS as being poor; she had earlier stated that she did not understand the instructions for scoring QUADAS. She also felt the studies in her review were of fairly poor quality but still fulfilled at least half the QUADAS items. All reviewers stated that they would use QUADAS again, although one stated that she may not use all 14 items next time and another stated that this was because there is currently no better tool available.

#### Additional comments

A major theme in reviewers' additional comments related to the poor quality of reporting of primary studies and the fact that this often limits the quality assessment. Another theme was that it is important to have an understanding of the clinical context while scoring some of the items. One reviewer suggested that it might be helpful to group the questionnaire using subheadings such as "general", "reference standard", and "index test". Another comment was that initial training on how to use the tool would be helpful.

## Discussion

### Principal findings

This evaluation has shown good agreement between reviewers and the final consensus rating for most QUADAS items and very positive feedback from reviewers who have used QUADAS. Two items, uninterpretable results and withdrawals, were found to be problematic. There was poorer agreement among reviewers and between reviewers and consensus for these items than for other items; feedback from reviewers also suggested problems with these items. One reviewer suggested that this might be because it is difficult to know what to do it if it is unclear if there are any uninterpretable results or withdrawals. Our own use of QUADAS supports this: we have found it very difficult to know how to score this item if the study does not report whether there were any uninterpretable results/withdrawals, and if all patients who entered the study appear to be accounted for. In such situations it is often unclear whether the study authors simply excluded uninterpretable results or withdrawals from their reports, or if there truly were no uninterpretable results or withdrawals. We have handled this problem by giving more explicit instructions for scoring these QUADAS items: we have stated that they should be scored as yes if it appears that all patients who were entered into the study completed the study.

The assessment of inter-rater reliability also highlighted possible problems with the items on the availability of clinical information and selection criteria. The item on clinical information is very specific to each review and it is therefore essential that clear guidelines on scoring this item be provided, outlining exactly what information should be available to the person interpreting the results of the index test. This definition should be agreed a priori. This was done for the review used for this evaluation and is reflected in the very high levels of agreement between two of the reviewers and the final consensus. It is unclear why the third reviewer showed much poorer agreement (50%) with the final consensus rating. It is unclear why the item on selection criteria showed poorer agreement with the consensus rating. This item was not highlighted as problematic in the feedback from reviewers. It may be related to the fact that no review specific information was provided for this item.

All additional items suggested for inclusion in QUADAS were considered as part of the development of QUADAS but were items that were not selected by the panel of experts for inclusion in the final tool. One of the items suggested for inclusion, the item relating to the threshold for the index test could be covered as part of item 8 (description of index test details). This is something to consider including in the guidelines for scoring this item when making guidelines specific to your review.

There was substantial variation in the time taken to complete QUADAS, ranging from less than 10 minutes to over 1 hour. This may be explained by the fact that some reviewers counted the time taken for the whole process of data extraction, including reading the paper, whereas others only counted the time taken to complete QUADAS. Despite this, half the reviewers took less than 15 minutes and 17/20 took less than half an hour to complete QUADAS suggesting that QUADAS is relatively quick to complete.

### Strengths and weaknesses of the study

The major strength of this study is that we carried out a detailed evaluation of QUADAS, which specifically included the views and experience of users. We are unaware of any other quality assessment tools for diagnostic accuracy studies that have undergone any process of evaluation.

Ideally, we would have liked to assess the "construct validity" of the tool – "the degree to which a test measures what it claims, or purports, to be measuring" [6]. As QUADAS aims to provide an indication of the quality of a study one way to assess this would be to take a set of "high" quality studies and a set of "low quality" studies and determine whether QUADAS can distinguish between these. This is known as "extreme groups" [6]. The problem with this process is determining which studies are high quality and which are low quality: there is no objective way of doing this. In addition, a systematic review is likely to include studies covering a range of quality. A quality assessment tool needs to be able to distinguish subtle differences across this full range of study quality, not just the extremes. We therefore decided against this method of evaluation.

### Unanswered questions and future research

We originally proposed to carry out a meta-epidemiological regression analysis to investigate the association of individual QUADAS items with estimates of test performance. However, due to limited time and resources such an evaluation was not feasible. This is an area where future research would be beneficial. The Cochrane Collaboration is planning to extend its database to include diagnostic test accuracy reviews and is in the process of producing a handbook providing guidelines for the conduct of such reviews. The recommendations on quality assessment include a modified version of QUADAS (items 2, 8 and 9, the items relating to reporting rather than quality have been removed), and this will be built into the new Cochrane software. All diagnostic reviews included in the new Cochrane Database will therefore include an assessment of QUADAS with the results entered into the Review Manager Software in a structured way. In the future, once a number of Cochrane Test Accuracy Reviews have been completed, a meta-epidemiological regression analysis can be pursued.

## Conclusions – Suggestions for modifications to QUADAS

We do not feel that major modifications to the content of QUADAS itself, in terms of items included, are necessary. However, the evaluation highlighted particular difficulties in scoring the items on uninterpretable results and withdrawals. We therefore recommend that the guidelines for scoring these items in the QUADAS background document be modified as shown in Table 4. In addition, we would like to highlight the importance of tailoring the guidelines for scoring items to each particular review, and of ensuring that all reviewers are clear on how studies should be scored for each of the items. It is not possible to provide a generic description of what should be considered an "appropriate patient spectrum", or what should be considered an "appropriate reference standard". It is therefore essential that all reviewers using QUADAS carefully consider how each individual item should be applied to their review and adapt the background document to make the guidelines for scoring specific to their review. This should be done in close collaboration with a clinical expert in the area of the review. Reviewers should also carefully consider whether all QUADAS items are relevant to their review, and also whether there are additional quality items not included in QUADAS which may be of importance to their topic area and which they should assess as part of their review. Consensus should be established on all of these issues before starting the quality assessment. Lastly, an improvement in the quality of reporting, by endorsing the standards for reporting of diagnostic accuracy studies, the STARD initiative [7], should occur. This will allow reviewers to assess study quality rather than the quality of reporting.

**Table 4:** Proposed modifications to the QUADAS background document*

**13. Were uninterpretable/intermediate test results reported?**
*c. How to score this item*
If it is clear that all test results, including uninterpretable/indeterminate/intermediate are reported then this item should be scored as "yes". **If the authors do not report any uninterpretable/indeterminate/intermediate results, and if results are reported for all patients who were described as having been entered into the study then this item should also be scored as "yes". **If you think that such results occurred but have not been reported then this item should be scored as "no". If it is not clear whether all study results have been reported then this item should be scored as "unclear".
**14. Were withdrawals from the study explained?**
*c. How to score this item*
If it is clear what happened to all patients who entered the study, for example if a flow diagram of study participants is reported, then this item should be scored as "yes". **If the authors do not report any withdrawals and if results are available for all patients who were reported to have been entered into the study then this item should also be scored as "yes". **If it appears that some of the participants who entered the study did not complete the study, i.e. did not receive both the index test and reference standard, and these patients were not accounted for then this item should be scored as "no". If it is not clear whether all patients who entered the study were accounted for then this item should be scored as "unclear".
* Proposed changes are highlighted in bold

*Each of these items were rated from 1–5 where 1 is strongly disagree (very poor) and 5 is strongly agree (excellent)

## Competing interests

The author(s) declare that they have no competing interests.

## Authors' contributions

PW, JK and PB conceived the study. All authors contributed to the design of the study. PW and MW collected the data. PW carried out the analysis and drafted the paper. All authors commented on drafts of the manuscript and read and approved the final manuscript.

## Pre-publication history

The pre-publication history for this paper can be accessed here:


